# Urolithin A prevents streptozotocin-induced diabetic cardiomyopathy in rats by activating SIRT1

**DOI:** 10.1016/j.sjbs.2021.09.045

**Published:** 2021-09-17

**Authors:** Gadah Albasher, Saad Alkahtani, Laila Naif Al-Harbi

**Affiliations:** a Department of Zoology, College of Sciences, King Saud University, Riyadh, Saudi Arabia; b Department of Food Science and Nutrition, College of Food and Agricultural Sciences, King Saud University, Riyadh, Saudi Arabia

**Keywords:** Urolithin A, Diabetic cardiomyopathy, Oxidative stress, SIRT1, Nrf2, NF-κB, Fibrosis, Rats

## Abstract

This study examined the cardiac anti-cardiomyopathy (DC) protective effect of urolithin A in streptozotocin (STZ)-treated rats and investigated if this protection involves activation of SIRT1 signaling. Diabetes was induced first STZ (65 mg/kg, i.p.) before starting the experiments. Adult male rats (n = 8/group) were treated for 8 weeks as control (non-diabetic), control + urolithin A (2.5 mg/kg/i.p.), STZ, STZ + urolithin A, and STZ + urolithin A + Ex-527 (1 mg/kg/i.p.) (a SIRT1 inhibitor). With no effect on fasting glucose and insulin levels, urolithin A improved left ventricular (LV) function and structure and reduced heart weight and serum levels of cardiac markers in STZ-treated rats. Also, it prevented collagen deposition, reduced mRNA levels of Bax, cleaved caspaspe3, collagen 1A1, transforming growth factor-β1 (TGF-β1), and Smad3 but enhanced those of Bcl2 in the LVs of diabetic rats. However, urolithin A suppressed the generation of reactive oxygen species (ROS), activated the nuclear factor erythroid 2–related factor 2 (Nrf2), and increased the levels of manganese superoxide dismutase (MnSOD) and total glutathione (GSH) in the LVs of the non-diabetic and diabetic rats, In parallel, it suppressed the cardiac activity of NF-nuclear factor-kappa beta p65 (κB p65) and reduced levels of tumor necrosis factor-α (TNF-α), and interleukin-6 (IL-6). Coincided with these events, urolithin A promoted higher activity, mRNA, and total/nuclear protein levels of SIRT1 and lowered the levels of acetyl-FOXO1, Nrf2, NF-κB, and p53. All these benefits of urolithin A were prevented by Ex-527. In conclusion, urolithin A protects against DC by activating SIRT signaling.

## 1 Introduction

Diabetic cardiomyopathy (DC) is the most common feature seen among diabetic patients (Jia et al., 2018). Currently, DC is a leading mechanism for the progression to heart failure (HF) and increased mortality among the affected patients (Miki et al., 2013, Jia et al., 2018). In general, DC is characterized by abnormalities in the structure and hemodynamic function of the heart, in the absence of any other cardiac disorder (Russo and Frangogiannis, 2016). However, adverse cardiac remodeling due to cardiomyocyte hypertrophy, inflammation, apoptosis, and fibrosis is the major mechanism underlies cardiac dysfunction in diabetic individuals and animal model (Russo and Frangogiannis, 2016, Jia et al., 2018, Jubaidi et al., 2020, Wang et al., 2020).

Despite the extensive research which was conducted recently, the molecular mechanisms underlying DC are still understudied and need further investigation. Up-to-date, increased production of reactive oxygen species (ROS) in the myocardium in response to hyperglycemia is the major key player in the pathogenies of DC in both types of diabetes mellitus (DM) and are the leading cause for activating numerous pathways involved in myocardial tissue remodeling (Ge et al., 2019, Ritchie and Abel, 2020, Jubaidi et al., 2020). These include downregulating the antioxidant transcription factor, the nuclear factor erythroid 2–related factor 2 (Nrf2), upregulation/activation of the inflammatory transcription factor, NF-nuclear factor-kappa beta p65 (κB p65), activation of the fibrotic pathway, the transforming growth factor (TGF-β1)/smad2/3, and promoting and the apoptotic cell death. However, suppressing oxidative stress by pharmacological antioxidants or overexpressing antioxidant enzymes attenuated hyperglycemia-induced hypertrophy, the impairment in cardiac contractility, and cardiac fibrosis (Thandavarayan et al., 2011, Ge et al., 2019, Ritchie and Abel, 2020).

Besides, DC alters the expression of several cellular survival factors in the heart. SIRT1, an NAD^+^ deacetylase, is the most-known survival molecule in most cells cardiomyocytes that stimulates survival and inhibit oxidative damage, inflammation, fibrosis, and apoptosis (Rahman and Islam, 2011, Morigi et al., 2018). The cellular effects afforded by SIRT1 are attributed to its ability to deacetylate numerous targets including NFκβ, Nrf2, forkhead box transcription factors (FOXO), the peroxisome proliferator-activated receptor-gamma coactivator (PGC1a), and p53 (Chong et al., 2012, Zhou et al., 2018). Of note, levels and activities of SIRT1 are significantly inhibited in the diabetic hearts and were linked to the severity of the cardiac damage and fibrosis (Sulaiman et al., 2010, Rizk et al., 2014, Bagul et al., 2015, Ma et al., 2017, Waldman et al., 2018, Zhang et al., 2018, Liu et al., 2019, Ren et al., 2020). In all these studies, pharmacological or transgenic activation of SIRT1 was always cardioprotective. Therefore, activation of SIRT1 seems a potential target to treat DC.

On the other hand, the interest in dietary modification and nutraceuticals to treat cardiovascular disorder by stimulating the deacetylation activity of SIRT1 has received substantial attention (De Boer et al., 2006). Urolithins are special gut metabolites that are products of the intestinal enzymatic hydrolysis of ellagic acid (EA) (Tang et al., 2017). In contrast to Ellagitannins (ETs) and EA, the bioavailability of urolithins is very high as being faster absorbed (Kim et al., 2020). After digestion, urolithins are can be found in different forms including urolithins A-D (Kim et al., 2020). Urolithin A is the most studied urolithins with many health benefits attributed to anti-inflammatory, anti-obesity, and antioxidant, potentials (Tang et al., 2017, Kim et al., 2020). The cardiac protective potentials of urolithin A were demonstrated in experimental animals of ischemia–reperfusion by upregulation of antioxidants (Tang et al., 2017). Also, administration of urolithin A alleviated streptozotocin (STZ)-mediated DC in rats where it preserved LV function hemodynamic parameters (Savi et al., 2017). However, the precise mechanism underlying the cardioprotective effect of urolithin A in diabetic hearts is poorly investigated. Of note, very recent evidence has shown that urolithin-A can prevent UV-induced DNA damage in keratinocytes, D-galactose-induced brain aging, and other models of renal damage, mainly, by upregulating and activating SIRT1 signaling (Chen et al., 2019, Djedjibegovic et al., 2020, Mohammed et al., 2020).

Overall, these findings were very encouraging and led us to hypothesize that urolithin A may alleviate STZ-induced DC by inhibiting cardiac oxidative damage, inflammatory response, intrinsic apoptosis, and fibrosis. Besides, we assumed that activation/upregulation of SIRT1 and the subsequent deacetylation of several targets (i.e FOXO1, Nrf2, NF-κB, and p53) mediate this protection.

## 2 Materials and methods

### 2.1 Animals

Male rats (strain: Wistar) (125 ± 15 g) were provided from the College of Sciences at King Saud University (KSU), Riyadh, Saudi Arabia. During the whole experiment period, all animals were kept in a private room in the animal house in a humidified environment (61%) with constant temperature (23 ± 1℃). All animal and experimental procedures were approved by the Research Ethics Committee at KSU (Ethical Reference No: KSU-SE-21–20).

### 2.2 Establishment of T1DM in rats

T1DM was introduced to the rats according to the protocol of Furman, 2015, Liu et al., 2020 using a single intraperitoneal injection of STZ (65 mg/kg) (Cat. No. Ab142155, Abcam, UK, Cambridge) freshly prepared in citrate buffer (pH 5.6). Three days post-STZ induction, rats with fasting hyperglycemia (fasting glucose > 350 mg/dl) were isolated and added in the experimental design as below. For this part, blood samples were collected from the rat’s tail (250 µl) and glucose levels were measured using a special assay kit (Cat. No. 10009582, Cayman chemicals, USA).

### 2.3 Urolithin preparations

Urolithin A powder (Cat. No. SML1791) and dimethyl sulfoxide (DMSO, Cat. No D2650) were purchased from Sigma Aldrich (MO, USA). Urolithin A was prepared at a concentration of 25 mg/ml DMSO and then diluted in PBS (pH = 7.4) to a concentration of 0.25 mg/ml (Savi et al., 2017). The final DMSO concentration in any treatment was 1%.

### 2.4 Experimental design

On day 3 after the induction of STZ and validating hyperglycemia, control (i.e. non-STZ-treated) and STZ-treated rats were categorized into 5 treatment groups (each of 8 rats): **1) control rats:** injected (i.p.) with an equal amount of 1% DMSO/PBS solution (as a vehicle); **2) control + urolithin A-treated rats:** control rats injected i.p. with the urolithin A solution (2.5 mg/kg); **3) STZ-T1DM rats:** diabetic rats and were treated i.p. with 1% DMSO/PBS solution (as a vehicle); **4) STZ + urolithin A-treated rats:** diabetic rats treated i.p. with the urolithin A solution (2.5 mg/kg); and 5**) STZ + urolithin A + EX-527-treated rats:** STZ-induced rats and were i.p. with concomitant injections of 2.5 mg/kg urolithin A solution and EX-527 (1 mg/kg) (a selective SIRT1 inhibitor). Treatments were given, daily, for consecutive 8 weeks. Any death in any group was replaced by others and underwent a similar treatment period. Body weights were taken every week and food intake was recorded every day.

### 2.5 Dose selection

The regimen of urolithin A was according to Savi et al. (2017) who showed a cardiac protective effect against the alterations in STZ-diabetic rats. The *in vivo* dose of EX-527 was selected for the study of Eid et al. (2021) who have shown that such concentration can maximally inhibit cardiac SIRT1 without any adverse effects.

### 2.6 Left ventricular (LV) hemodynamic measurement

For this part, we have followed the study of Eid et al. (2021). The measurements were based on measuring LV pressure using a pressure catheter (model SPR 320, Millar, USA) and recording and analyzing the signal using a PowerLab data acquisition system (Model No 16/35, ADInstruments, Sydney, Australia). In brief, by the end of the experiment, each rat of every group was fasted for 8 h and then anesthetized (80/12 mg/kg ketamine/xylazine mixture, i.p.). During the experimental measurements, the temperature of each rat was maintained by a heating pad and monitored using an anal thermal probe. Besides, both eyes were kept moist using a gel ointment. A small incision was made to expose the left carotid artery (LCA) which was freed from the surrounding nerves and tissues. After that, the Millar catheter, connected to the bridge amplifier and the PowerLab recorder, was inserted through the LCA and directed to the LV. The pressure signal was recorded for 20 min. The first 5 min were not included in the analysis and were considered as an adaptation period. The data of the last 15 min was analyzed using the pressure signal module on the associated LabChart software (Version 8, ADInstruments, Sydney, Australia) to calculate the various pressure-related parameters including LVSP: LV systolic pressure; LVEDP: LV end-diastolic pressure; dP/dtmax: the maximum derivative of the change in systolic pressure over time; and dP/dtmin: the minimum derivative of the change in diastolic pressure.

### 2.7 Blood and tissue collection

Directly, after the hemodynamic measurement, a 1 ml blood sample was collected in either EDTA-supplied or empty tubes. All tubes were centrifuged for 10 min at 1200*g*. Plasma and serum samples were separated into new tubes and maintained at −20℃. Then, all rats were ethically authenticated and the hearts were rapidly removed, weighted. Then all LVs were isolated on ice. The LV of each rat was cut into smaller parts. Some parts were placed in formalin solution and used for the routine histological study (described below). All other pieces of each LV were preserved at −80℃ for further usage.

### 2.8 Preparation of cell homogenates and the nuclear/cytoplasmic fractions

Ice-cold phosphate-buffered saline (PBS) was used to homogenate the total tissue for the biochemical analysis. The radioimmune precipitation assay (RIPA) buffer was used to homogenate the tissue and preparing the total protein extract for western blotting. In brief, LV (50 mg) were individually, and respectively suspended and homogenized in 9 volumes (450 µl) PBS (pH = 7.4) or 1X radioimmune precipitation assay (RIPA) buffer (plus protease inhibitor), homogenized and centrifuged (12,000*g*/10 min/4℃). The supernatants containing total cell homogenates or total proteins were separated and preserved at −70℃ until use. Also, the nuclear/cytoplasmic fractions were prepared from other LV tissues using the provided kit (Cat. No. 113474, Abcam, Cambridge, UK). Always, proteins concertation in any given sample was determined using the supplied kit (Cat. No. 23225; Thermo-Fisher Scientific).

### 2.9 Biochemical analysis in the plasma and determination of HOMA-β

Glucose levels were evaluated using the provided kit (Cat No. 81693; Crystal Chem, IL, USA). Insulin levels were evaluated using ELISA kits (Cat. No. MBS2700141, MyBioSource, CA, USA). The values of the HOMA-β (i.e. hemostatic model assessment of β-cell function) was determined as described by Zadjali et al. (2012) (HOMA-IR = [fasting insulin (ng/mL) × 20]/[fasting glucose (mg/dL) – 3.5]). All measurements were performed as per the instructions of the kits.

### 2.10 Serum analysis

Serum levels of creatinine kinase-MB (CKMB) and Troponin-I (Tn-I) were measured with the help of commercially available kits as per the manufacturers’ instructions (Cat. No. MBS2515061 and Cat. No. MBS727624, MyBioSource, CA, USA, respectively).

### 2.11 Biochemical measurement in LV homogenates

The content of ROS was evaluated using an assay kit (Cat. No. STA-347, Cell Biolabs, CA, USA). The concentration of the total glutathione (GSH), malondialdehyde (MDA), manganese superoxide dismutase (MnSOD), tumor necrosis alpha (TNF-α), and interleukin-6 (IL-6) were *analyzed* by ELISA (Cat No. MBS046356, Cat. No. MBS268427, Cat No. MBS729914, Cat. No. MBS175908 and Cat. No MBS175904; MyBioSource, CA, the USA respectively). All measurements were performed as per each kit's instructions.

### 2.12 Biochemical analysis in the nuclear fractions

The activities of SIRT1 in the nuclear isolates were determined using a fluorometric kit (Ab165065, Abcam, Cambridge, UK). All protocols were conducted as per each kit's instructions. All measurements were performed as per each kit's instructions.

### 2.13 Real-time PCR

qPCR was used to assess the mRNA levels of serval targets in the LV tissue of all groups of rats. The primers used for the amplification were designed on the available online software and were all purchased from ThermoFisher (Table 1). TRIzol reagent (ThromoFisher Scientific, USA) was used to prepare the total RNA. A commercially available cDNA synthesis kit was used to synthesize the template first-strand cDNA (Cat. No. 11754050, ThromoFisher scientific, USA). The amplification reaction was performed with the help of a Bio-Rad CFX96 thermal cycler using the Ssofast Evergreen Supermix 1725201 (Bio-Rad, Montreal, Canada) as per the manufacturer's instructions. PCR amplification was conducted in a 96 well plate of 20 µl reaction containing the following: 500 ng/µl cDNA (2 μl), 200 nM primers (0.4 μl of 10 Μm of each primer), Ssofast Evergreen Supermix (10 μl), and RNase-free water (7.2 μl). PCR reactions were: an initial enzyme inactivation (95℃/30 sec/1 cycle), denaturation (95℃/5 sec/40 cycles), annealing (60 ℃/60 sec/40 cycles), and melting (95℃/30/1 cycle). All targets were normalized β-actin (a reference gene) using the ΔΔCT method. Two negative controls were included in each run. All measurements were performed as per each kit's instructions.

**Table 1:** List of the primers utilized in the qPCR reaction.

Target	number	F primer	R primer	bp
SIRT1	XM_003751934.1	TGTTTCCTGTGGGATACCTGA	TGAAGAATGGTCTTGGGTCTT	137
NF-κB1	XM_342346.4	AATTGCCCCGGCAT	TCCCGTAACCGCGTA	157
TGF-β1	NM_021578.2	GGCGGTGCTCGCTTTGTA	GGGTGACTT CTTTGGCGTAG	104
Smad3	NM_013095.3	5′-GGGCCTGCTGTCCAATGT-3′	5′-AATGTGCCGCCTTGTAAGCT-3	112
COLI	NM_053304	TTCACCTACAGCACGCTTGT	TGGGATGGAGGGAGTTTAC	196
Nrf2	NM_031789.1	TTTGTAGATGACCATGAGTCGC	TGTCCTGCTGTATGCTGCTT	142
HO-1	NM_012580.2	TCTGCAGGGGAGAATCTTGC	TTGGTGAGGGAAATGTGCCA	135
Bcl2	U34964.1	TGGGATGCCTTTGTGGAACT	TCTTCAGAGACTGCCAGGAGAAA	73
Bax	NM_017059	ATGGAGCTGCAGAGGATGATT	TGAAGTTGCCATCAGCAAACA	97
Caspase-3	U49930	AATTCAAGGGACGGGTCATG	GCTTGTGCGCGTACAGTTTC	60
Β-actin	NM_031144.2	TACCCAGGCATTGCTGACAG	AGCCACCAATCCACACAGAG	115

### 2.14 Western blotting

Total and nuclear, and cytoplasmic proteins were suspended in the loading dye at a concentration of 2 µg/kg. Equal protein concentrations from all samples were loaded and separated on different percentages of SDS-PAGE and transferred to nitrocellulose membranes. After blocking with the 5% skimmed milk, all membranes were washed and then incubated with the 1st antibody (2 h at room temperature) against SIRT1 (Cat. No. 9475, Cell Signalling Technology, USA, 120 kda/1:000), Cytochrome-c (Cat. No. 11940, Cell Signaling Technology, USA, 14 kDa, 1:1000), Acetyl Nrf2 (Lys599) (Cat. No. STJ9230, St John's Laboratory Ltd, London, UK, 61 kDa/1:500), NF-κB p65 (Cat. No. 8242, Cell Signaling Technology, USA, 65 kDa, 1:1000), Acetyl NF-κB (Lys310) (Cat. No. PA5-17264, ThermoFisher, USA, 65 kDa/1:1:500), Acetyl-FOXO1 (Lys294) (Cat. No. PA5-104560, ThermoFisher, USA, 70 kDa/1:500), Acetyl p53 (Lys382) (Cat. No. 2525, Cell Signalling Technology, USA, 53 kDa, 1:1000), Nrf2 (Cat. No. sc-365949, Santa Curz Biotechnology, 61 kDa, 1/500), α Tubulin (Cat. No. sc-5286, Santa Cruz Biotechnology, USA, 55 kDa), β-actin (Cat. No. 4970, Cell Signaling Technology, USA, 45 kDa/1:1000), lamine A (Cat. No. 86846, Cell Signaling Technology, USA, 69 kDa, 1:1000). Next, the membranes were incubated with the corresponding 2nd antibody for 2 h at room temperature. 1x tris-buffered saline, 0.1% Tween 20 (TBST) buffer was used to dilute the antibodies and preparing the blocking milk. All washing steps were conducted using the TBST buffer as 3 washes, each of 10 min. Bands detection was performed using 5 min incubation with the chemiluminescence ECL reagents A and B (Cat. No. 32109, ThermoFisher, USA, Piscataway, NJ). Band intensities on all gels were analyzed using the C-Di Git blot scanner. Lamin A was used as a nuclear reference protein whereas β-actin of the total proteins.

### 2.15 Histological evaluation

All samples were deparaffinized and rehydrated in Xylene, 100%, 95%, and 70% ethanol, and deionized water. The samples were then stained with Harris hematoxylin with glacial acetic and rinsed with deionized and tap water. Distaining was achieved by incubating the slides with acid ethanol. The slides were then washed with tap water and stained with eosin Phloxine stain. Then, all samples were dehydrated with 95% and 100% ethanol and xylene. A drop of permuting media was added to each tissue and then covered with a coverslip for overnight drying. The images were captured under a light microscope at 200×.

### 2.16 Statistical analysis

The GraphPad Prism statistical software, version 8, was used to analyze and graph the data. The Kolmogorov-Smirnov test was used for normality testing. Analysis was done by the 2-way ANOVA. Tukey’s *t*-test was used as the post-hoc test. P values < 0. 05 were considered significantly varied.

## 3 Results

### 3.1 Changes in some metabolic markers, hemodynamic function, and cardiac markers

While fasting plasma insulin levels, the final body weights, and HOMA-β were significantly decreased, fasting plasma glucose, heart weight, heart index, and serum levels of CKMB and Troponin-I showed a significant increase in the STZ-treated group in comparison with the control (Table 2**)**. Also, a significant reduction in LVSP, dp/dt_max_, and dp/_dtmin_ were observed in the STZ-treated group (Table 2). The levels of these LV function parameters and cardiac markers, HOMA-β, as well as plasma glucose and insulin levels, were not different when the control + urolithin A-treated group as compared to the control group (Table 2). However, while rat’s final body weights, fasting glucose and insulin levels, and levels of HOMA-β1 were not significantly different, heart weight, heart index, serum levels of CKMB and Troponin-I, and values of LVEDP were significantly reduced, and levels of LVSP, dp/dt_max_, and dp/dt_min_ were significantly increased in STZ + urolithin A-treated rats as compared to STZ-treated rats (Table 2). Of note, no significant variations in the levels of all these measured targets were seen between the STZ + urolithin A + EX-527-treated rats and STZ-treated group, thus suggesting that EX-527, a SIRT1 inhibitor, prevented the effect of urolithin A on these markers (Table 2).

**Table 2:** Heart weights, glucose, and insulin levels, cardiac function, and cardiac enzymes levels in all groups of rats.

Parameter	Control	Control + urolithin A	STZ	STZ + urolithin A	STZ + urolithin A + EX-527
Final body weight (g)	378 ± 15.6	381 ± 22.2	276 ± 11.2^ab^	287 ± 14.3^ab^	273 ± 17.4^ab^
Heart weights (g)	1.04 ± 0.12	0.99 ± 0.18	1.38 ± 0.16 ^ab^	1.12 ± 0.13^c^	1.29 ± 0.11^abd^
H/BW ratio (×10^−3^)	2.58 ± 0.45	2.61 ± 0.52	3.62 ± 0.58^ab^	3.59 ± 0.67^ab^	3.66 ± 0.49^ab^
Fasting glucose (mg/dl)	98.6 ± 8.6	96.7 ± 7.3^a^	311 ± 15.4^ab^	321 ± 18.7^ab^	318.4 ± 22.4^ab^
Fasting insulin (ng/ml)	2.1 ± 0.43	2.3 ± 0.65	0.53 ± 0.13^ab^	0.49 ± 0.15^ab^	0.58 ± 0.11^ab^
HOMA-β (×^10−2^)	42 ± 6.2	49 ± 11.1	3.4 ± 0.61^ab^	3.9 ± 0.59^ab^	3.6 ± 0.71^ab^
CK-MB (pg/ml)	124 ± 12.4	132 ± 10.4	598 ± 12.4^ab^	198 ± 13.1^abc^	635 ± 37.7^abd^
Troponin-I (pg/ml)	96.7 ± 8.5	89.4 ± 7.8	468 ± 22.1^ab^	148 ± 11.3^abc^	478 ± 19.3^abd^
LVSP (mmHg)	114 ± 10.9	109 ± 13.4	71.4 ± 8.5^ab^	103 ± 7.5^c^	65.6 ± 7.5^abd^
LVEDP (mmHg)	3.5 ± 0.61	3.73 ± 0.49	13.54 ± 1.8^ab^	4.61 ± 0.71^abc^	15.2 ± 3.5^abd^
Dp/dt_max_ (mmHg/s)	6773 ± 344	6219 ± 413	3221 ± 192^ab^	5029 ± 216^abc^	2992 ± 281^abd^
Dp/dt_min_ (mmHg/s)	5019 ± 409	5311 ± 492	2691 ± 319^ab^	4837 ± 344^c^	2525 ± 317^abd^

For each group (n = 8), the data are given as mean ± SD. Levels of significance were considered at *p* < 0.05. **^a^**: compared to control group; **^b^**: compared to the control + urolithin A; **^c^**: compared to STZ, **^d^**: compared to STZ + urolithin A.

### 3.2 Changes in LVs, collagen deposition, and fibrotic markers

**Fig. 1:**
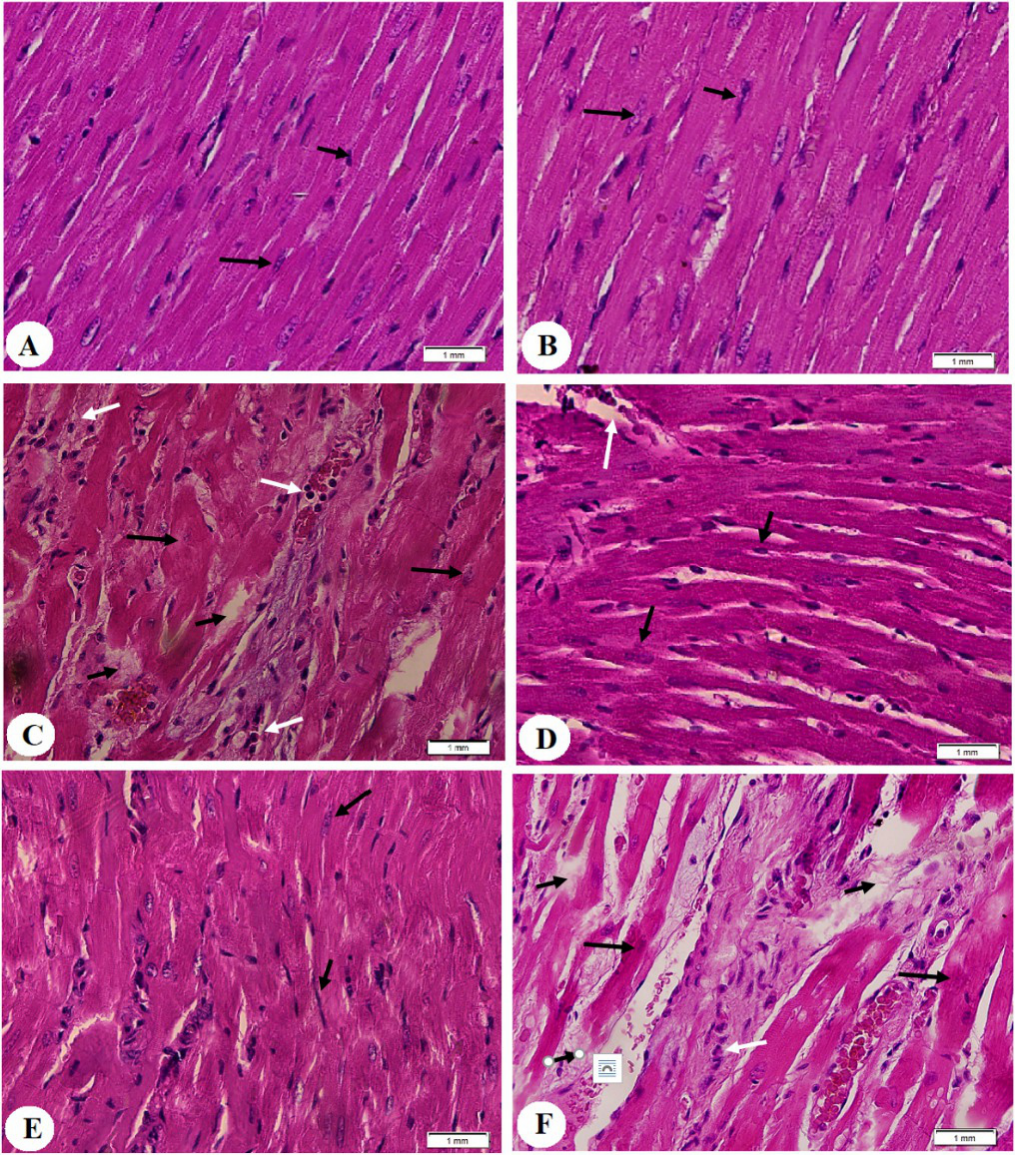
**Histological images obtained from the left ventricles (LVs) of all groups of rats (H&E stain200x). *A&B:*** were taken from control and control + urolithin A-treated rats and showed normal cardiac fibers with intact central nuclei (long black arrow) and peripheral endothelial cells (short black arrow). ***C:*** was taken from an STZ-induced rat and showed deeply stained hypertrophied cardiomyocytes with karyolytic nuclei (long black arrow), increased cardiomyocyte damage (short black arrow), and increased inflammatory cell infiltration (white arrow). ***D and E:*** were taken from STZ + urolithin A-treated rats and showed almost normally sized cardiomyocytes with normally appeared intact nuclei (long black arrow) and endothelial cells (short black arrow). However, some damage in the cardiomyocytes is still seen (white arrow). ***F:*** was taken from an STZ + urolithin A + EX-527-treated rats and showed a mirror image of the changes observed in STZ-induced rats.

**Fig. 2:**
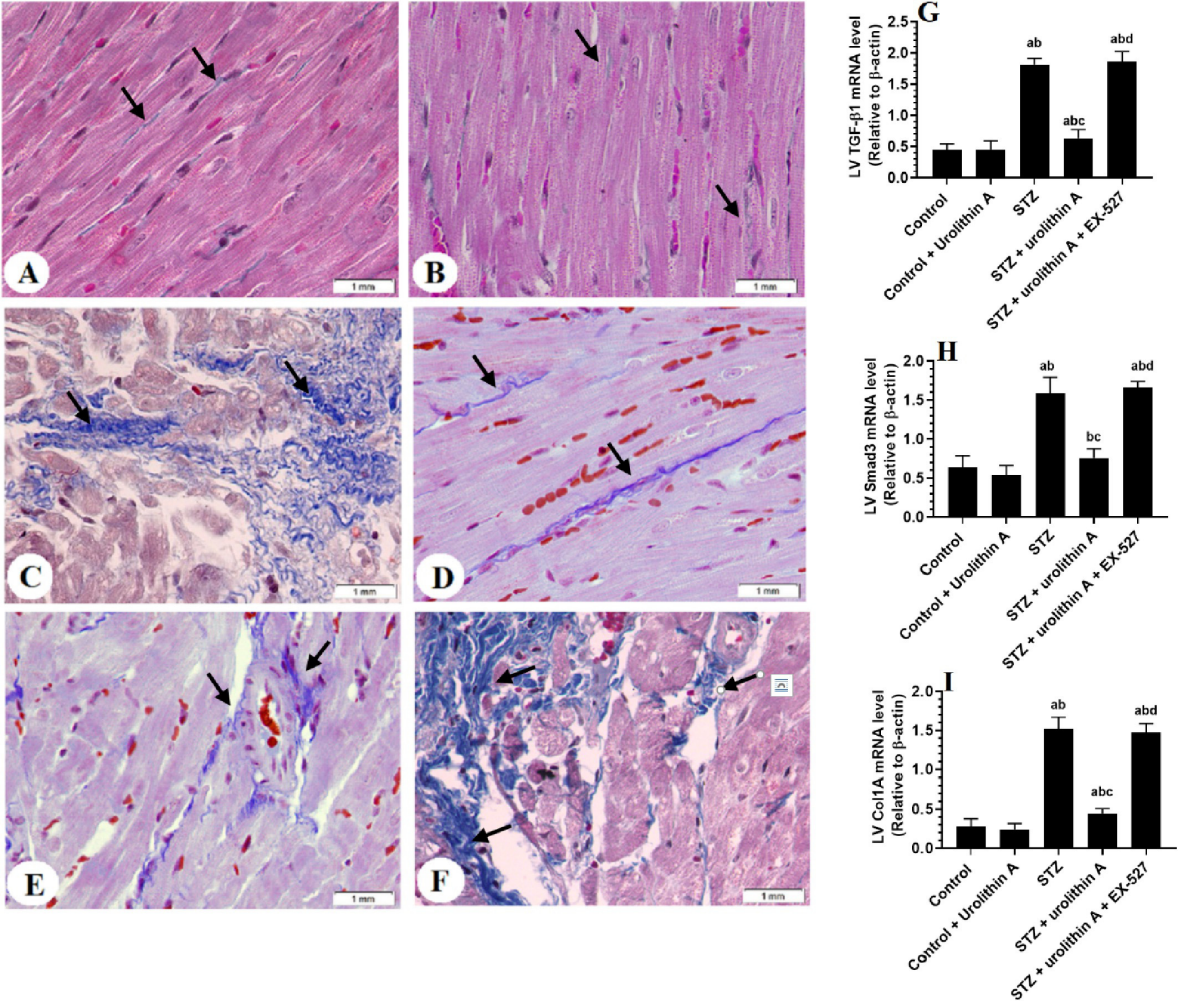
**Photomicrographs showing collagen deposition (A-F), as well as mRNA levels of TGF-β1 (H), Smad3 (H), and collagen 1A1 (Col1A1) (I) in the left ventricles (LVs) of all experimental groups. the degree of fibrosis was detected using the Masson’s trichrome stain (blue coloration) (200x). *A&B:*** were taken from control and control + urolithin A-treated rats and showed very few amounts of collagen fibers (black arrows) ***C:*** was taken from an STZ-induced rat and showed increased deposition of collagen fibers (black arrows. ***D and E:*** were taken from STZ + urolithin A-treated rats and showed reduced amounts of collagen fibers as compared to STZ-induced rats (black arrow). ***F:*** was taken from an STZ + urolithin A + EX-527-treated rat and showed increased collagen fibers deposition as compared to STZ + urolithin-treated rats. **In G-I:** For each group (n = 8), the data are given as mean ± SD. Levels of significance were considered at *p* < 0.05. **^a^**: compared to control group; **^b^**: compared to the control + urolithin A; **^c^**: compared to STZ, **^d^**: compared to STZ + urolithin A.

Administration of urolithin A to control rats didn’t affect the cardiomyocyte structure or the number of collagen fibers nor the LV mRNA levels of Col1A1, Smad2, and TGF-β1 in comparison to the control group (Fig. 1 **A&B,** Fig. 2 **A&B and** Fig. 2 **G-I).** However, STZ-treated rats showed disorganized hypertrophied deeply stained muscle fibers with obvious damage in their nuclei and structure and increased inflammatory cell infiltration in their myocardium (Fig. 1**C)**. Also, the LVs of this group of rats had an increased amount of deposited collagen (Fig. 2**C)** and significantly higher mRNA levels of TGF-β1, Smad2, and Col1A1 as compared to the control group (Fig. 2**G-I).** On the contrary and as compared to the STZ-treated group, normal LV architectures with less collagen deposition, and lower levels of mRNA of all these targets were seen observed in the LVs of STZ + urolithin A-treated group of rats (Fig. 1 **D&E,** Fig. 2 **D&E and** Fig. 2**G-I).** Like the STZ-treated group, the LVs of STZ + urolithin A + EX-527-treated group of rats had damaged myocardium, increased collagen deposition, and higher mRNA expression of TGF-β1, Smad2, and Col1A1 (Fig. 1**F,** Fig. 2**F,** Fig. 2 **G-I).**

### 3.3 Changes in markers of oxidative stress/antioxidants

**Fig. 3:**
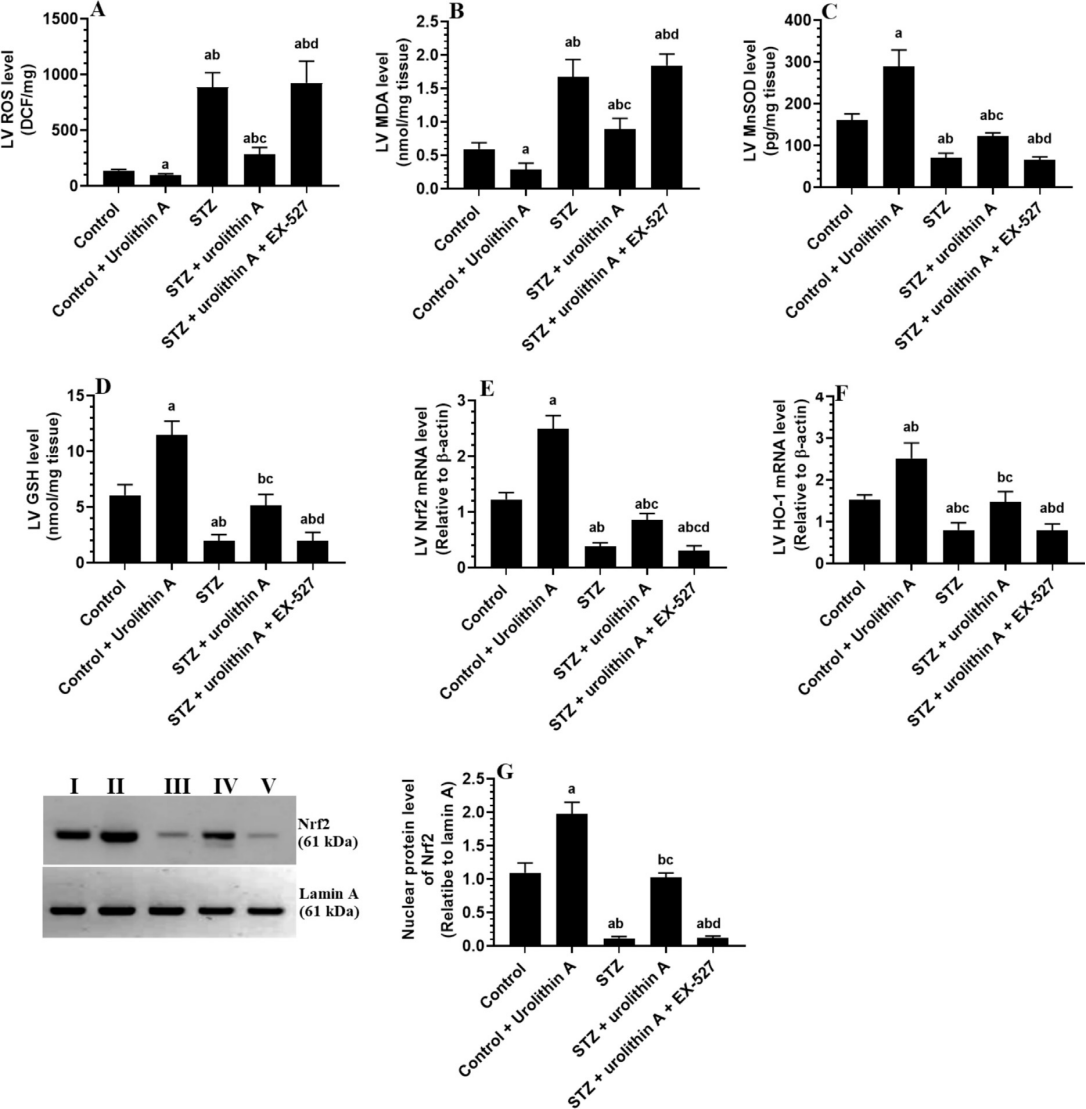
**Levels of ROS (A), MDA (B), MnSOD (C), and GSH (D), mRNA levels of Nrf2 € and HO-1 (HO-1), and nuclear protein levels of Nrf2 in the LVs of all experimental groups.** For each group (n = 8), the data are given as mean ± SD. Levels of significance were considered at *p* < 0.05. **^a^**: compared to control group; **^b^**: compared to the control + urolithin A; **^c^**: compared to STZ, **^d^**: compared to STZ + urolithin A.

LVs of the STZ-treated group of rats had higher cardiac levels of ROS and MDA and showed significantly lower levels of GSH and MnSOD, mRNA of Nrf2 and HO-1, and nuclear protein levels of Nrf2 as compared to control non-diabetic rats (Fig. 3**A-G).** However, administration of urolithin A to either the control (control + urolithin A) or diabetic rats (STZ + urolithin A) significantly reduced levels of ROS and MDA and stimulated the levels of GSH and SOD, as well as mRNA levels of Nrf2 and HO-1, and nuclear protein levels of Nrf2 in their LVs as compared to the control or STZ-treated group of rats, respectively (Fig. 3**A-G).** Similar not significant changes in the levels/expression of all these oxidative stress/antioxidant markers were observed when STZ + urolithin A + EX-527-treated group was compared to STZ-diabetic rats (Fig. 3**A-G).**

### 3.4 Changes in inflammatory markers

**Fig. 4:**
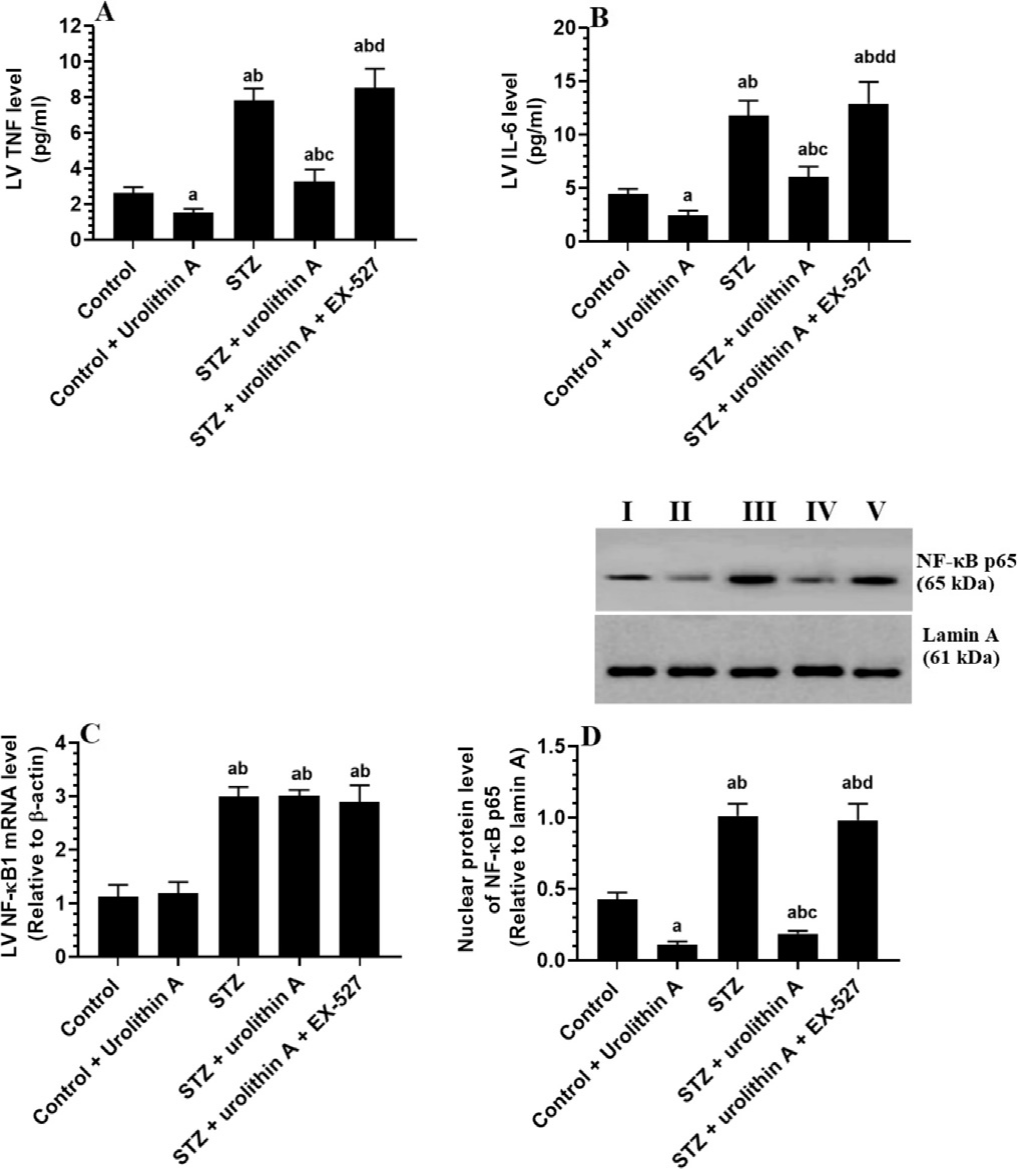
**Levels of TNF-α and IL-6 (B) (mRNA levels of NF-κB1 (C), and nuclear protein levels of NF-κB p65 in the LVs all of all** experimental **groups.** For each group (n = 8), the data are given as mean ± SD. Levels of significance were considered at *p* < 0.05. **^a^**: compared to control group; **^b^**: compared to the control + urolithin A; **^c^**: compared to STZ, **^d^**: compared to STZ + urolithin.

LVs of rats of the STZ-induced group had significantly higher levels of TNF-α and IL-6 and showed increased mRNA and nuclear protein levels of NF-κB as compared to control non-diabetic rats (Fig. 4**A-D)**. Control + urolithin A and STZ + urolithin A-treated groups showed no significant change in the cardiac mRNA levels of NF-κB but had a significant reduction in levels of TNF-α and IL-6 and nuclear protein levels of NF-κB p65 as compared with the control or STZ-group (Fig. 4**A-D).** However, as compared to the STZ + urolithin A-treated group, levels of TNF-α and IL-6, and protein levels of NF-κB p65 but mRNA levels of NF-κB were not significantly altered in the LVs of STZ + urolithin A + EX-527-treated group (Fig. 4**A-D).** No variations in the level of all endpoints were seen between STZ-induced rats and STZ + urolithin A + EX-527-treated rats (Fig. 4**A-D).**

### 3.5 Changes in markers of intrinsic cell apoptosis

**Fig. 5:**
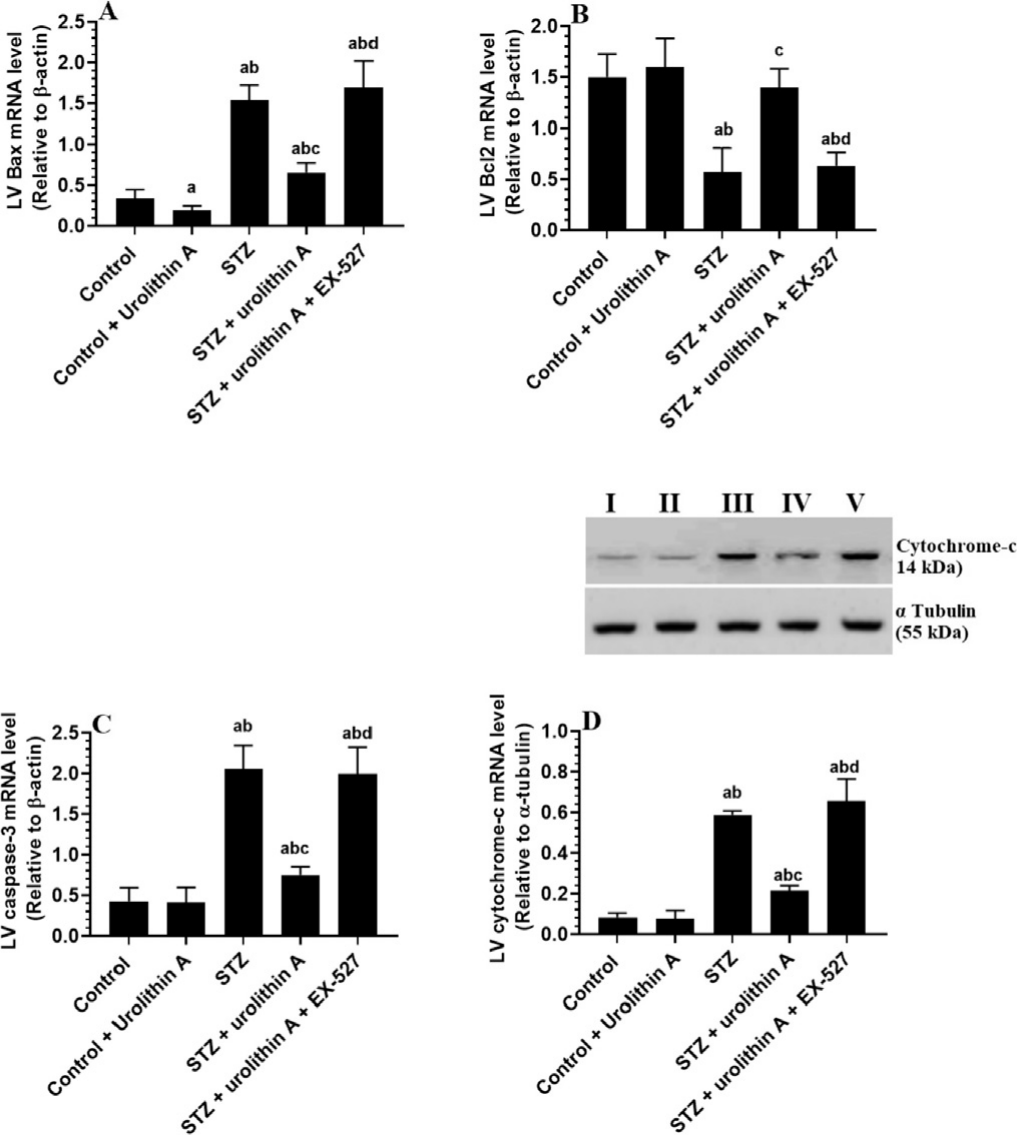
**mRNA levels of Bax (A), Bcl2 (B), and caspase-3 (C), as well as the cytoplasmic level of cytochrome-c (D) in the LVs of all experimental groups.** For each group (n = 8), the data are given as mean ± SD. Levels of significance were considered at *p* < 0.05. **^a^**: compared to control group; **^b^**: compared to the control + urolithin A; **^c^**: compared to STZ, **^d^**: compared to STZ + urolithin.

LVs of the control + urolithin A-treated group of rats had significantly less mRNA levels of Bax compared to the control group (Fig. 5**A-D)**. LVs of STZ-model rats showed a significant increase in the cytoplasmic protein levels of cytochrome-c and mRNA levels of Bax and cleaved caspase-3, with a coincided decrease in the mRNA of Bcl2 in comparison to the control group (Fig. 5**A-D)**. On the contrary, mRNA levels of Bax and cleaved caspase-3, as well as the protein levels of cytochrome-c were significantly reduced but protein levels of Bcl2 have significantly increased in the LVs of STZ + urolithin A-treated rats as compared to STZ-induced rats, an effect which were completely reversed in STZ + urolithin A + EX-527-treated rats (Fig. 5**A-D)**. Upon analysis, the levels of all these apoptotic/anti-apoptotic markers were similar between STZ-induced rats and STZ + urolithin A + EX-527-treated rats (Fig. 5**A-D)**.

### 3.6 Changes in activity and expression of SIRT1

**Fig. 6:**
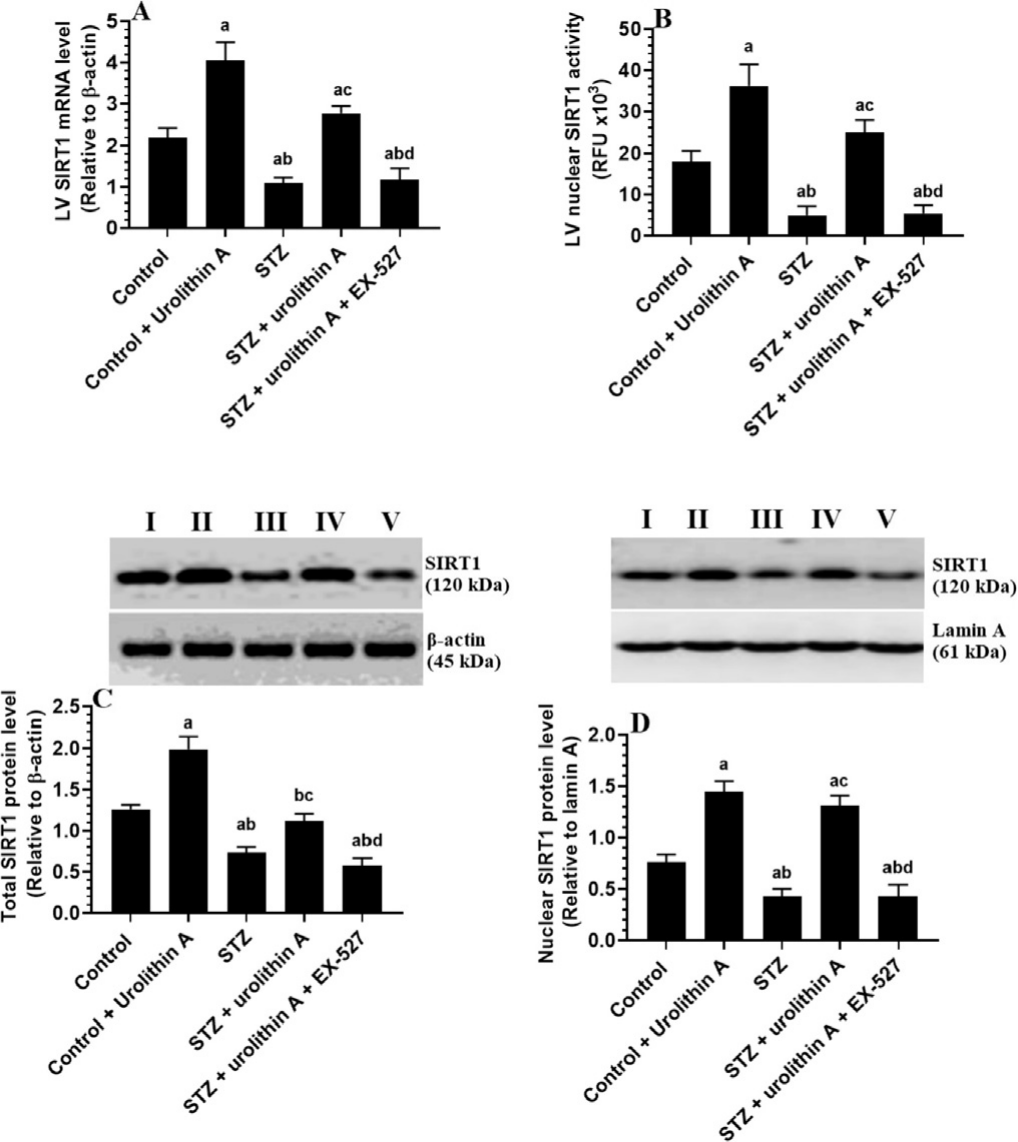
**mRNA (A) nuclear activity (B), total protein (C), and nuclear protein (D) level of SIRT1 in the LVs all of all experimental groups.** For each group (n = 8), the data are given as mean ± SD. Levels of significance were considered at *p* < 0.05. **^a^**: compared to control group; **^b^**: compared to the control + urolithin A; **^c^**: compared to STZ, **^d^**: compared to STZ + urolithin.

LVs of the STZ-model group of rats showed significantly lower mRNA levels, nuclear activity, total protein, and nuclear protein levels of SIRT1 as compared to the control group (Fig. 6**A-D)**. Significantly increased mRNA, nuclear activity, and total/nuclear protein levels of SIRT1 were seen in the LVs of both the control + urolithin A and STZ + urolithin A-treated groups as compared to either the control or STZ-model groups, respectively (Fig. 6 **A-D)**. However, administration of EX-527 to STZ + urolithin-treated rats significantly reduced the mRNA, nuclear activity, and nuclear/total protein levels of SIRT1 as compared to STZ + urolithin A–treated rats, levels which were not significantly varied when compared to STZ-model rats (Fig. 6 **A-D)**.

### 3.7 Changes in the acetylation of some transcription factors

**Fig. 7:**
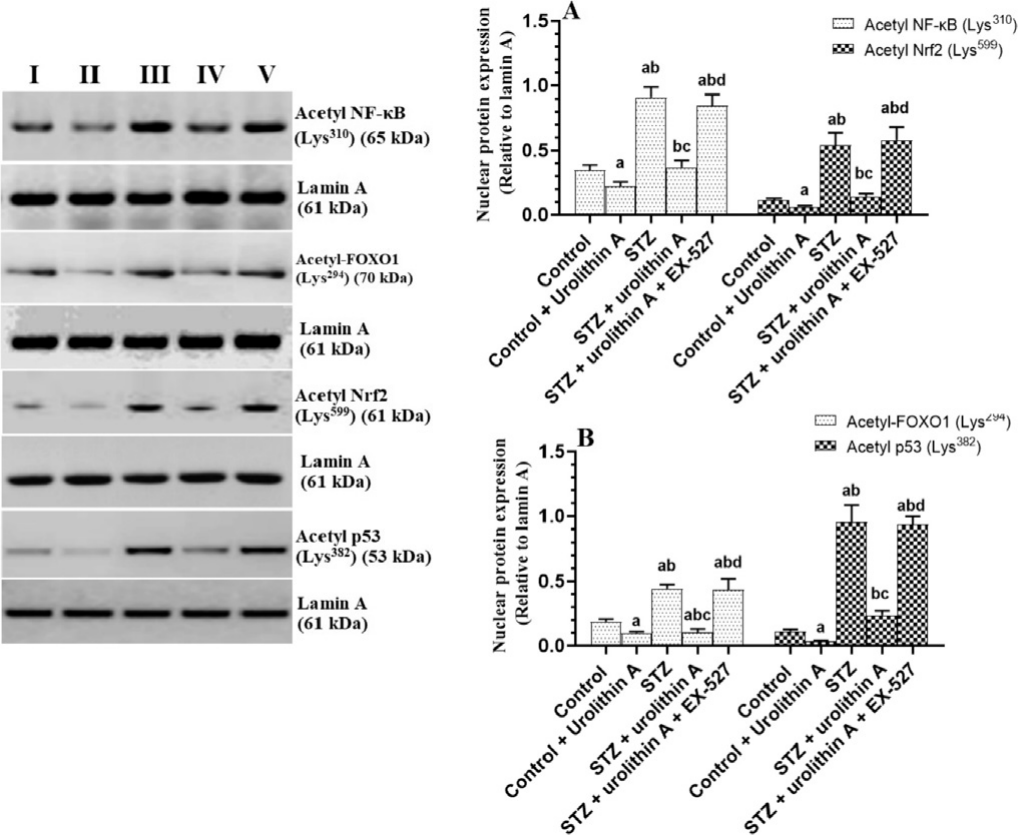
**Acetylated protein levels of NF-κB (A), Nrf2 (B), FOXO1 (B), and p53 (D) in the LVs all of all experimental groups.** For each group (n = 8), the data are given as mean ± SD. Levels of significance were considered at *p* < 0.05. **^a^**: compared to control group; **^b^**: compared to the control + urolithin A; **^c^**: compared to STZ, **^d^**: compared to STZ + urolithin.

Acetylated protein levels of Nrf2, NF-κB, p53, and FOXO1 were significantly increased in the nuclear extracts of the LVs of STZ-treated rats as compared to control rats (Fig. 7 **A&B)**. However, nuclear-acetylated protein levels of all these transcription factors were significantly decreased in the LVs of both the control + urolithin A and STZ + urolithin A-treated rats as compared to the control or STZ-treated rats, respectively (Fig. 7**A&B)**. On the other hand, acetylated protein levels of Nrf2, NF-κB, p53, and FOXO1 were significantly higher in the LVs of STZ + urolithin A + Ex-527 as compared to STZ + urolithin A-treated rats but not significantly different as compared STZ-treated rats (Fig. 7**A&B)**.

## 4 Discussion

The two polyphenols, Ellagitannins and ellagic acid (EA) exert many health benefits against several chronic disorders (Espín et al., 2013). However, both Ellagitannins and EA have poor absorption rates and many of their biological effects were attributed to the generation of urolithins which can inhibit oxidative stress, inflammation, hypoglycemia, and tumorogenesis (Espín et al., 2013). During the last decade, the cardioprotective effect of urolithin A has been confirmed against I/R-induced cardiac injury and DC in rodents (Tang et al., 2017). Indeed, urolithin A was able to reduce the infarct size and attenuate LV apoptosis (Tang et al., 2017). However, in the STZ-diabetic hearts, the protection afforded by urolithin A was attributed to its potential to restore normal cardiac hemodynamic function and suppressing inflammatory cell infiltration and cardiomyocyte inflammation, as well as regulating the activity of some intracellular Ca^2+^ hemostasis-related proteins, thus leaving the molecular mechanism largely unknown (Savi et al., 2017).

In this study, our inserting data showed that urolithin A can be classified as a SIRT1 activator in the rat’s heart that can stimulate SIRT1 transcription, translation, and activation. Accordingly, through upregulating and activating SIRT1, our data indicate that urolithins A are indispensable for the acetylation of Nrf2, NF-κB, p53, and FOXO1 transcription factors, which mediates its biological protective effects including upregulating of the cardiomyocyte endogenous antioxidants and inhibiting oxidative stress, inflammation, fibrosis, and apoptosis. Besides, these data were supported by the observation that *in vivo* suppressing of SIRT1 by the specific inhibitor, EX-527, abolished all the cardiac effects afforded by urolithin A.

DC is characterized by LV stiffness which results in impaired heart function due to overproduction of ROS and activation of cardiac remodeling (Tan et al., 2020). The key player in this situation is hyperglycemia and the subsequent huge amount of generation of ROS which subsequently induce cardiac damage and remodeling (Kotturu et al., 2020, Tan et al., 2020). The mechanism by which hyperglycemia induces ROS in the heart is well-reported and involves several sub-mechanisms including the direct activation of the glucose-dependent oxidant pathways (i.e. PKC, advanced glycation end product (AGEs), and polyol and hexosamine pathways), damaging the mitochondria, impairing the process of oxidative phosphorylation and Ca^2+^ handling, activating the renin-angiotensin system (RAS), stimulating ROS generating enzymes (i.e. NADP oxidase and CaMKII, and downregulating/inhibiting Nrf2 (Kaludercic and Di Lisa, 2020, Lu et al., 2020, Jubaidi et al., 2020, Tan et al., 2020).

Nevertheless, ROS can induce damage in the cardiomyocytes and induces inflammation and intrinsic cell (mitochondria-mediated) apoptosis by promoting lipid peroxidation, inducing DNA brakes and upregulating p53, activating p53/Bax apoptotic axis, downregulating anti-apoptotic genes (i.e. Bcl2), damaging the mitochondria (cytochrome-c release), and activation inflammatory markers including NF-κB, NLRP3 inflammasome, and TNF-α (Jia et al., 2018, Chen et al., 2020, Tan et al., 2020). Together, hyperglycemia, ROS, and TNFα, and NF-κB induce cardiac hypertrophy and fibrosis by activating the fibroblasts and the TGF-β1/Smad2/3 signaling (Fiordelisi et al., 2019, Russo and Frangogiannis, 2016). Therefore, currently available studies support that controlling hyperglycemia by pharmacological insulin administration or any other hypoglycemic agent and/or suppressing ROS are the best therapeutic strategies to prevent DC in diabetic individuals or animals (Jia et al., 2018).

In this study, the obvious amelioration in the systolic and diastolic function, the reduction in the circulatory cardiac enzyme levels, and the improvement in the cardiomyocytes structure with the concomitant reduction in collagen deposition were our first guide for the protective effect of urolithin A against the DC in the STZ-treated rats. These data support the findings of other authors (Savi et al., 2017) who also shown that administration of urolithin A at a similar dose improved the impairment in the LV hemodynamic function in STZ-induced rats. However, our data dissipate the hypoglycemic or insulin modulatory effect of urolithin A from its cardioprotective effect since we couldn’t observe any changes in the glucose and insulin homeostasis and rats’ body weights in both the non-diabetic and STZ-treated rats. On the other hand, our data support that such cardioprotective effect of urolithin A is being rather mediated by other cardiac mechanisms which may involve antioxidant, and anti-inflammatory potentials. These data oppose those observed by Tuohetaerbaike et al. (2020) who have demonstrated a potent ability of urolithin A to exert hypoglycemic and insulin-releasing effects through prompting pancreatic protective autophagy in HFD + STZ-induced T2DM rats. Such variations between their study and ours could be explained by the difference in the animal model used (T2DM vs. T1DM), STZ dose (85 mg/kg vs. 65 mg/kg), the dose of urolithin A (50 mg/kg vs. 2.5 mg/kg), and time of urolithin A and administration (direct at the time of STZ injection vs. three days later). Indeed, Bayle et al. (2019) have reported that different urolithins can stimulate insulin secretion from the pancreatic cells, in vitro, but this was dose-dependent where urolithin C being the most effector.

Indeed, the data of this study assure that the anti-remodeling potential of urolithin A is mediated by scavenging ROS, upregulation, and activation of Nrf2, stimulating antioxidants, inhibiting the nuclear activation of NF-κB, and downregulating Bax. Indeed, administration of urolithin A to the all treated rats (i.e. control plus diabetic) stimulated the transcription of Nrf2 and HO-1, stimulated the transactivation of Nrf2, increased levels of SOD and GSH, inhibited the nuclear activation of NF-κB, reduced levels of the measured cytokines, and downregulated Bax. However, since urolithin A didn’t affect the nuclear content of NF-kB but reduced its nuclear accumulation in the hearts of both treated groups, it seems very reasonable to us that the anti-inflammatory potential of urolithin A is due to suppressing NF-κB and subsequent reduction in the generation of TNF-α and IL-6. However, urolithin A only suppressed TGF-β1 and Col1A1 in the LV of STZ-treated rats only. As mentioned above, ROS, NF-κB, TNF-α are potent stimulators of TGF-β1 (Fiordelisi et al., 2019, Russo and Frangogiannis, 2016). These data indicate that the inhibitory potential of urolithin A on this fibrosis is secondary to its antioxidant and anti-inflammatory effects.

Supporting our data, urolithins A attenuated cardiac damage after I/R through downregulation of keap1 and subsequent upregulation and activation of Nrf2 (Zheng et al., 2020). In the same way, urolithins A prevented apoptosis and reduced the infarct size in I/R exposed heart by upregulating antioxidants (Tang et al., 2017). Also, urolithins A stimulated endogenous antioxidants expression inhibited the production of ROS and suppressed inflammation in other tissues of several animal models such as liver cancer, neurodegeneration, atherosclerosis, ulcerative colitis; cisplatin-induced nephropathy, carrageenan-induced paw edema, etc. (Djedjibegovic et al., 2020). In this view, several authors have shown that urolithins A stimulates the cellular antioxidant ability by numerous mechanisms including scavenging ROS; chelating iron, upregulation of Nrf2 and antioxidants (i.e. GSH and other enzymes like SOD, GPx, catalase, peroxiredoxins), inhibiting ROS generating pro-oxidant peroxidases [i.e. myeloperoxidase (MPO), lactoperoxidase (LPO), and phorbol myristate acetate (PMA)], suppressing mitochondria ROS generation, and possessing antiglycation activity (Verzelloni et al., 2011, Ishimoto et al., 2012, Saha et al., 2016, Komatsu et al., 2018, Mazumder et al., 2019, Cásedas et al., 2020, Djedjibegovic et al., 2020, Lee et al., 2021). Besides, urolithin A prevents cellular inflammation by suppressing NF-κB signaling and downregulating the synthesis of prostaglandin-E2 and COX-2 (Komatsu et al., 2018, Xu et al., 2018, Fu et al., 2019).

Nonetheless, an interesting finding in this study is that the cardioprotective and anti-fibrotic potentials of urolithins A against STZ-induced DC are associated with upregulating the transcription and the translation, as well as increase the nuclear localization and activation of SIRT1 which resulted in subsequent deacetylation of Nrf2, FOXO1, NF-κB, and p53. In general, SIRT1 deacetylates FOXO3 and thus leading to an increase in the resistance to oxidative stress by upregulating MnSOD and other antioxidant targets (Olmos et al., 2013). Besides, SIRT1 promotes the activation of Nrf2 to stimulate the synthesis of SOD and GSH and downregulate TGF-β1 (Singh et al., 2018, Ren et al., 2019). Also, SIRT1 deacetylates the p65 subunit to suppress the transcriptional activity of NF-kB (Kauppinen et al., 2013, Ren et al., 2019). Furthermore, and not least, SIRT1 can stimulate the expression of MnSOD and catalase and suppresses the transcription of many apoptotic factors such as CDKNIA and BAX by deacetylating p53 (Cheng et al., 2014, Gonfloni et al., 2014, Rahman and Islam, 2011).

As urolithin A stimulated the cardiac levels and the activation of SIRT1 in the diabetic and non-diabetic animals, these data may provide a reasonable reason for the mechanism by which urolithin A-induced suppression of NF-κB, transactivation of NRf2, and downregulation of Bax in the hearts of these rats. Besides, these data indicate that the antioxidant abilities of urolithin A are due to the deacetylation of FOXO1, Nrf2, and p53 whereas the anti-inflammatory could be attributed to deacetylating of NF-κB. To support our hypothesis, we have suppressed SIRT1 by EX-527 which prevented all the cardioprotective and anti-fibrotic effects of urolithin A that was coincided with increased acetylation of all the above-mentioned transcription factors.

Nevertheless, although our data are the first to show a possible regulation of urolithin A on cardiac levels of SIRT1 in diabetic animals, many previous studies support our findings. In this regard, ellagic acid (EA) was shown to protect against iron-induced kidney damage in rats by activation of SIRT1 (Mohammed et al., 2020). Also, urolithin A augmented angiogenic pathways in skeletal muscle, prevented D-galactose-induced brain aging, prevented UV-induced DNA damage in the keratinocytes, and suppressed oxidant damage in the microglial and neural progenitor cells by stimulating SIRT1 (Chen et al., 2019, Ghosh et al., 2020).

Despite this, we still have some limitations. Accordingly, our data is still observation and further future studies using SIRT1 knockdown animals or cell cultures with the use of some inhibitors that suppresses the transcription or the translation of SIRT1 such as actinomycin D and cycloheximide could confirm our data. Besides, further studies targeting other related pathways such as AMPK, a major inducer of SIRT1 are highly recommended. Also, searching further mechanisms that can be activated after urolithin A administration which can regulate SIRT1 expression and activation (i.e. AMPK) needs further investigation.

In conclusion, the current study confirms the protective role of urolithin A against STZ-induced DC in rats. The data presented in this study support our hypothesis and confirm that such a protective effect is mediated, at least, by activating SIRT1 expression and deacetylase activity. These data encourage the use of urolithin A in future clinical studies in diabetic and other disease conditions which require the activation of SIRT1.

## Declaration of Competing Interest

The authors declare that they have no known competing financial interests or personal relationships that could have appeared to influence the work reported in this paper.
